# Impact of the transition to digital pathology in a clinical setting on histopathologists in training: experiences and perceived challenges within a UK training region

**DOI:** 10.1136/jcp-2022-208416

**Published:** 2022-07-29

**Authors:** Lisa Browning, Lucinda Winter, Rosalin A Cooper, Abhisek Ghosh, Thomas Dytor, Richard Colling, Eve Fryer, Jens Rittscher, Clare Verrill

**Affiliations:** 1 NIHR Oxford Biomedical Research Centre, Oxford, UK; 2 Cellular Pathology, Oxford University Hospitals NHS Foundation Trust, Oxford, UK; 3 Cellular Pathology, Wycombe Hospital, High Wycombe, UK; 4 Nuffield Department of Clinical Laboratory Sciences, University of Oxford, Oxford, UK; 5 Nuffield Department of Surgical Sciences, University of Oxford, Oxford, UK; 6 Department of Engineering Science, University of Oxford, Oxford, UK; 7 Big Data Institute, University of Oxford, Oxford, UK

**Keywords:** education, pathology department, hospital, pathology, surgical, diagnosis

## Abstract

**Aims:**

With increasing utility of digital pathology (DP), it is important to consider the experiences of histopathologists in training, particularly in view of the varied access to DP across a training region and the consequent need to remain competent in reporting on glass slides (GS), which is also relevant for the Fellowship of the Royal College of Pathologists part 2 examination. Understanding the impact of DP on training is limited but could aid development of guidance to support the transition. We sought to investigate the perceptions of histopathologists in training around the introduction of DP for clinical diagnosis within a training region, and the potential training benefits and challenges.

**Methods:**

An anonymous online survey was circulated to 24 histopathologists in training within a UK training region, including a hospital which has been fully digitised since summer 2020.

**Results:**

19 of 24 histopathologists in training responded (79%). The results indicate that DP offers many benefits to training, including ease of access to cases to enhance individual learning and teaching in general. Utilisation of DP for diagnosis appears variable; almost half of the (10 of 19) respondents with DP experience using it only for ancillary purposes such as measurements, reporting varying levels of confidence in using DP clinically. For those yet to undergo the transition, there was a perceived anxiety regarding digital reporting despite experience with DP in other contexts.

**Conclusions:**

The survey evidences the need for provision of training and support for histopathologists in training during the transition to DP, and for consideration of their need to maintain competence and confidence with GS reporting.

**WHAT IS ALREADY KNOWN ON THIS TOPIC**

- While there is an understanding of the need for guidance for histopathologists transitioning to reporting clinical cases with digital pathology, guidance specifically to address the needs of histopathologists in training is not well established.

**WHAT THIS STUDY ADDS**

- Assessment of the experiences of histopathologists in training within a region with access to digital pathology provides new understanding of their perceptions of the benefits and challenges of adoption of digital pathology and their specific training needs.

**HOW THIS STUDY MIGHT AFFECT RESEARCH, PRACTICE OR POLICY**

- The survey results highlight the need to consider histopathologists in training during the transition to digital pathology, and to ensure a means to develop their confidence in its utility within the diagnostic setting. We outline key considerations for training in digital pathology and the potential support needed for those working within training regions with variable access to digital pathology.

## Introduction

Adoption of digital pathology (DP) within clinical practice is in the early stages in the UK. There are currently few centres with a fully digital set-up for diagnostic reporting; however, other centres are beginning the transition. The benefits (perceived and actual) and the challenges for diagnostic pathology practice are well documented;^1 2^ however, there are limited data about the acceptance and the impact of DP on histopathology training. As one of the first UK centres to undertake the transition to diagnostic DP,^3^ we previously explored the considerations necessary for histopathologists in training (hereafter ‘trainees’) in relation to this, and what measures might be beneficial in support of their specific training needs,^4^ proposing a programme of training with a theoretical and practical introduction ahead of DP transformation.

As the transition to DP continues to evolve nationally and internationally, there is now comprehensive literature on DP validation for diagnostic reporting^5 6^ and increasing numbers of experiential commentaries on DP implementation,^2 7–12^ with national and international groups being established to support the pathology community in the transition, and onward to the development and use of artificial intelligence (AI).^13–16^ However, similar support for trainees is lacking.

Uniquely, we have been able to reflect on the trainees’ experience of the introduction of diagnostic DP to our region. Through an online survey, we have explored their opinions regarding the transition to diagnostic digital reporting, and the wider potential benefits of DP to training and education, and any perceived challenges that access to DP introduces.

## Methods

A survey was circulated via the online SurveyMonkey survey tool (www.surveymonkey.com) to 24 trainees in their first year of training and above, based in four regional hospitals: the index tertiary referral centre which has been fully digitised since summer 2020^3^ and three district general hospitals, one of which is currently partially digitised and the others without a digital diagnostic service.

The survey results would be used to inform training in DP within the region, and through better understanding of the reported perceptions and opinions, potentially improve the success of both DP training and utilisation.

The survey comprised 33 individual questions (online supplemental file), about demographics, personal experience of DP within a clinical diagnostic setting, wider experience of DP for education and in other settings, current level of access to diagnostic DP, perceived training needs in relation to the transition to DP, impact of DP on histopathology training and examinations, and for those without current DP experience in a clinical setting there were questions on perceived readiness for transition, and areas of perceived potential benefit and challenge in the use of DP in the diagnostic setting. There were also questions on attitudes and opinions to both DP and AI in histopathology. The trainees were asked to consider their responses in relation to DP experience in the absence of the impact of COVID-19, as far as possible.

10.1136/jcp-2022-208416.supp1 Supplementary data

## Results

Nineteen trainees responded to the survey (79% response rate) and all answered in full. Respondents were at varying stages in their training, with 11 of 19 in the first 3 years of training, and 8 of 19 in their 4th year or above. In relation to examination status (Fellowship of the Royal College of Pathologists, FRCPath), 5 of 19 were post-FRCPath part 2 (all five with experience of diagnostic DP), with the remainder pre-FRCPath part 2 (9 of 19 pre-FRCPath part 1).

### General level of experience of DP

Eleven of 19 trainees reported that they had worked in a centre with access to DP for diagnosis, although only 10 of 19 had personal experience in using DP for diagnosis, of whom 9 of 19 had >6 months of DP experience. One respondent with DP experience for diagnosis in one hospital (>6 months) had since moved to a new post in a hospital without current access to DP.

A minority (3 of 19) had exposure to DP during undergraduate medical education in histology or histopathology.

**Figure 1:**
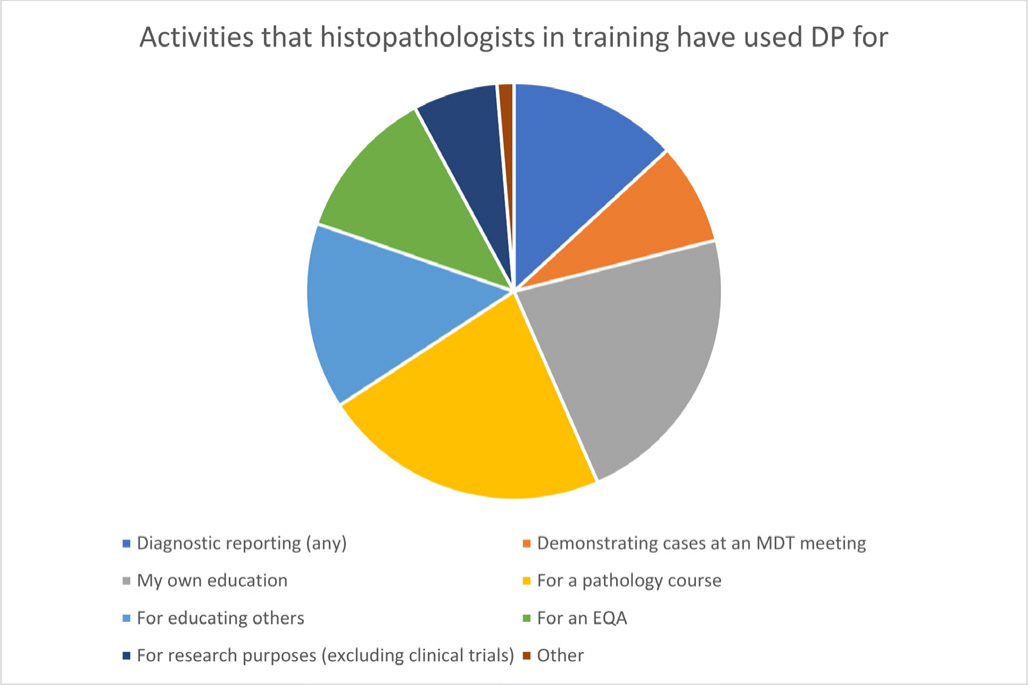
Activities that histopathologists in training have used digital pathology (DP) for to date. EQA, external quality assurance scheme; MDT, multidisciplinary team.

All respondents had experience of DP in at least one context (figure 1), and the majority (17 of 19) had used online digital slide resources for their own education or in the context of a course, and over half (11 of 19) had used DP slides for educating others.

Ninety per cent (9 of 10)using DP for diagnosis were aware of the Royal College of Pathologists (RCPath) guidance on the implementation of digital pathology;^17^ however, only 33% (3 of 9) working outside the department using DP were aware of this guidance.

### Training to use DP in the diagnostic setting

Seven of 19 reported having not received any specific training in the use of DP for diagnosis, including 20% of those currently using DP for diagnosis. Almost half had attended a training day on DP (8 of 19), and 3 of 19 had received training from the DP vendor (including 30% of those currently using DP). Validation sets collated in-house for consultant pathologists undergoing DP validation had been reviewed by 2 of 19 respondents, both with experience of diagnostic reporting on DP. No respondents had undertaken a formal ‘validation’ process.

### Experience with DP in the diagnostic setting

All trainees who have been based in the centre with routine diagnostic DP had experience in reporting surgical pathology digitally (10 of 19), and in the use of DP for sharing diagnostic cases flagged by colleagues for educational purposes. The extent to which these trainees currently use DP for diagnostic work is variable; 4 of 10 (3 of whom are post-FRCPath part 2) report using DP only for specific aspects of diagnostic reporting, such as assessment of measurements (tumour, margins), and prefer to report on glass slides (GS). The remaining 6 of 10 review both digital slides and GS for each case, with 4 of 6 reviewing *all* GS for a case (as opposed to review of selected GS). None report solely on DP. In terms of confidence in DP reporting, 6 of 10 do not feel confident but believe that they will do with additional experience. Of those who were more confident, the time taken to gain confidence was very variable (<1–>6 months).

Specific considerations related to diagnostic DP experience and perceived differences between DP and GS reporting are presented in table 1.

**Table 1:** General opinions of histopathologists in training in relation to reporting diagnostic cases on digital images versus GS

	Strongly disagree	Disagree	Neutral	Agree	Strongly agree
I am confident in making diagnoses on DP (generally)	0/10	2/10	2/10	5/10	1/10
I do not feel that DP for clinical diagnosis is different to diagnosis on GS	0/10	3/10	6/10	1/10	0/10
I find reporting on DP more difficult (generally) than on GS	1/10	3/10	3/10	3/10	0/10
It takes me longer to review diagnostic cases digitally compared with glass (in general)	0/10	4/10	2/10	1/10	3/10
When reporting a case, I usually review the digital slides in preference to the GS	0/10	7/10	1/10	2/10	0/10
I prefer reporting digitally to reporting on GS	1/10	6/10	2/10	1/10	0/10
I am concerned about the accuracy of reporting on digital images vs GS	0/10	3/10	3/10	4/10	0/10
I have awareness of recognised areas of potential pitfall in diagnosis on DP in general	0/10	0/10	1/10	8/10	1/10
I find that reviewing cases on the diagnostic digital platform (IMS) is superior in terms of ability to make a diagnosis, to reviewing a similar case on a non-diagnostic platform (ie, for educational use)	0/10	1/10	2/10	4/10	2/10

DP, digital pathology; GS, glass slides; IMS, information management system.

An essential part of training is the opportunity to review diagnostic cases with an experienced colleague, typically the consultant pathologist responsible for sign-out of the diagnostic report. DP introduces additional considerations around this, which have been impacted further by the COVID-19 pandemic and the requirement for social distancing and remote working. In our institution, there has been an overlap between the introduction of DP and the safe working requirements of the pandemic which are difficult to disentangle. However, pandemic aside, trainees reporting diagnostic cases on DP have various options for reviewing cases with a consultant colleague:

- DP together in real time in the same location.
- DP together in real time but remotely via videoconferencing.
- Review of cases separately with email communication about the findings.
- Trainee review of case on DP but then on GS in real time with the consultant.

Respondents did not express a preference as to which of these case sharing options was most beneficial to training. Six of 10 agreed that it made no difference whether the case was co-reviewed on GS or digitally, although case sharing limited to email communication was least favoured.

Trainees were asked to comment (free text) on areas they found to be easier with DP and those they felt to be more challenging (table 2).

**Table 2:** Areas identified by histopathologists in training as a potential challenge with the digital platform and areas which may potentially be made easier

Areas of potential challenge with the digital platform	
Diagnostic considerations	Lack of nuclear detail (4 comments) Assessing mitotic count (2 comments) Depth of focus/3D assessment (2 comments) Fear of missing something, including small details (2 comments) Time taken to assess the digital slides (2 comments) Grading of tumour Identifying microorganisms Refractile material
Technical considerations	Image lagging Out-of-focus images Reproduction of colours Digital system failure Waiting for slides to be scanned
**Areas that may potentially be easier with the digital platform**	
Diagnostic considerations	Making measurements (9 comments) Low power view including megablocks (6 comments) Comparing slides (eg, H&E with immunohistochemistry, 3 comments) Easier to ensure all of the slide is seen Easier to visualise the whole case that is for review Identification of lymphovascular invasion Assessment of small gastrointestinal biopsies, polyps, appendix, gall bladders
Workflow considerations	Sharing of cases with colleagues Recall of slides for a prior related case
Patient care-centred considerations	Reduces risk of misplacing or breaking slides
Teaching/training related	Annotating images to share with colleagues and at MDT meetings (6 comments)

3D, three-dimensional; MDT, multidisciplinary team.

### Training to report diagnostic cases on a digital platform

Those trainees using DP for diagnostic reporting were asked about training in relation to this. While they generally felt supported in the transition to DP, 4 of 10 felt that they had not received sufficient training to report digitally (with 4 of 10 neutral on this point). Only 1 of 10 felt that specific training was not necessary in relation to making the transition. Four of 10 were concerned about maintaining competence in reporting on GS.

The following specific areas were agreed as being of interest for future training, in order of popularity: potential challenges/pitfalls in digital diagnosis, data governance and ethical considerations, use of digital platform (functionality), integration of DP into the laboratory workflow and information technology considerations.

### General considerations around the wider impact of access to diagnostic DP on training experience

**Figure 2:**
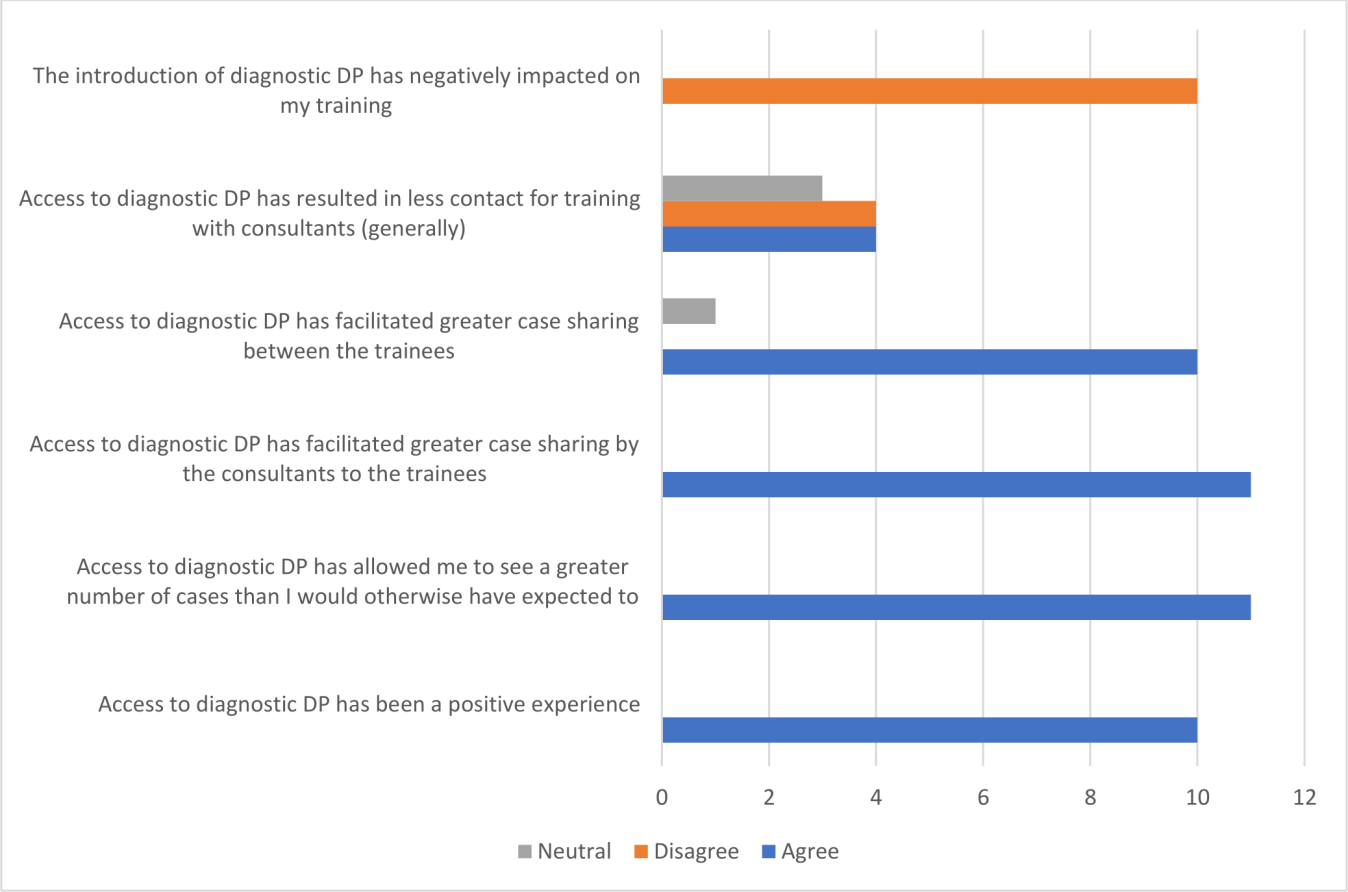
Opinions of histopathologists in training around the wider impact on training experience of the access to diagnostic DP (in the digitally enabled centre). Note that 11 respondents have answered some questions (additional respondent without diagnostic DP experience). DP, digital pathology.

For those within the DP-enabled centre, all agreed it had been a positive experience, facilitating generally improved case sharing and access to cases (see figure 2). None felt that the introduction of DP had negatively impacted their training.

Free-text comments included:

- ‘Reduction of pressure on trainees who can keep slides but consultant can review digital images and order extra work in parallel.’
- ‘…overall the introduction of DP is positive in that it makes a huge number of cases accessible and reviewable to trainees, however DP and the option for more remote working has meant that I feel I had less contact with consultants and time going through cases.’

In relation to readiness for the FRCPath examinations, it is noteworthy that no respondents felt that using DP for diagnosis would impact examination preparation negatively, although 1 of 19 felt that the largely digital nature of cases shared for teaching sessions in region might have a negative impact. Overall, DP was seen to facilitate access to a greater range and numbers of cases than otherwise might be expected, with 12 of 19 and 13 of 19 agreeing this to be the case, respectively (the remainder being neutral or unsure).

Given the nature of the FRCPath part 2 examination, a case-based practical assessment set on GS, we focused specifically on any perceived potential impact of DP on the examination preparation. Five of 19 have undertaken the FRCPath part 2 since DP was introduced within region, and the opinions of these trainees were split:

- One of five felt that reporting and reviewing cases regularly on DP (vs GS) has an impact (negative) on readiness for the examination.
- Two of five (three of five neutral) preferred to report on GS during examination preparation.
  - Mixed view as to whether it would have been more helpful to review cases on GS with a consultant during this time.

While there was no preference as to whether teaching cases were more helpfully seen on GS versus DP in the examination preparation period, three of five and two of five agreed that practice examinations and FRCPath part 2 courses (respectively) were better on GS versus DP (the remainder being neutral). Subjectively, the respondents regarded access to DP (including remotely) as having made examination preparation easier overall.

Free-text comments in relation to DP and the examinations included:

The issue for FRCPath examinations is there is a tendency to neglect exam practice cases on glass especially for cytology.

Whilst it is better to see cases on glass slides in preparation for the exam, being able to have regular black box sessions and access to essentially a ‘library’ of cases digitally is much more time-efficient. This benefit completely outweighs the cost of seeing ‘less’ glass to a degree.

….I would not be happy to sit a digital Part 2 examination without formal training and significant clinical experience with DP.

### Perceptions of trainees in non-DP-enabled training centres within region

While this had been explored to an extent in our previous work,^4^ the current survey provides a novel insight specifically as to how *variable* access within the region may itself impact on perceptions in contrast to investigation of perceptions of a DP-naïve cohort. The results are presented in table 3.

**Table 3:** Perceptions of histopathologists in training working in non-digital centres of the impact of variability of DP access within region

	Strongly disagree	Disagree	Neutral	Agree	Strongly agree	Don’t know	N/A
I am concerned that the variability in access to DP for diagnosis across training centres will impact negatively on my training overall	0/19	1/19	0/19	5/19	2/19	0/19	11/19
I do not consider the variability in access to DP across training centres to be an issue in relation to my training experience in histopathology	1/19	6/19	1/19	0/19	0/19	0/19	11/19
I feel that access to DP within any centre within the training region has had a positive impact on my training overall	0/19	0/19	0/19	3/19	2/19	1/19	13/19
I feel that DP is overhyped	1/19	4/19	0/19	3/19	0/19	0/19	11/19
I am apprehensive about the transition to DP in my own practice when this is available to me	1/19	1/19	2/19	1/19	3/19	0/19	11/19
I look forward to being able to report diagnostic cases on a digital platform	0/19	0/19	1/19	3/19	0/19	4/19	11/19
I do not feel that reporting diagnostic cases on a digital platform will be any different to reporting on glass slides	1/19	6/19	0/19	1/19	0/19	0/19	11/19

DP, digital pathology; N/A, not applicable.

It is evident from these results that this cohort of trainees feels that access to DP anywhere within region has a positive impact on their training overall (including five of six in non-digital centres), although there are clear concerns about the variable access to diagnostic DP within region and to their own transition to DP reporting.

Notably, one respondent had moved from a centre with access to DP and experience with digital reporting to one without access. This doctor suggested that the transition could be challenging, in view of changes in workflow and access to cases, and the loss of benefits of DP such as ease of measurements and low power assessment, although overall did not perceive negative impact on training.

### Perception of the impact of availability of DP on training and future job prospects, and on the promise of AI

Overall, there was positivity in relation to the impact on training of the introduction of DP into the region, with 84% (16 of 19) agreeing that this has been a positive experience, and 89% (17 of 19) agreeing that it has provided greater training opportunities. Considering impact on their future consultant careers, 95% (18 of 19) agree that the ability to report on both DP and GS will be beneficial, and 74% (14 of 19) agree that personal experience with diagnostic DP during training will impact on future job choices (more likely to apply for jobs with access to DP).

Finally, while the survey has not focused on AI, there was good awareness of the potential role for AI in pathology, with almost all respondents having awareness of the potential for AI to aid diagnosis, prognostication and derivation of novel insights into disease. Seventy-four per cent (14 of 19) look forward to the potential of using AI, although around one-third (7 of 19) remain concerned about the potential for DP and AI to replace pathologists. Thirty-seven per cent (7 of 19) have already had involvement in research in the development of AI, and overall, 79% (15 of 19) would like to be involved.

## Discussion

The literature on the transition to the use of DP for diagnostics is extensive; however, training and guidance specifically related to histopathologists in training are rarely mentioned, and the opinions and perceptions of those in training to the transition to DP are largely unknown.

In contrast to the governance structures in place for consultant histopathologists in terms of developing and ensuring competence in diagnostic reporting on whole slide images, including RCPath guidance on a formal validation process to reporting digitally,^17^ and guidance from the College of American Pathologists,^18^ there is currently no such equivalent for trainees nor recommendation within these documents as to how they might be considered during DP implementation, although awareness exists that such guidance is warranted.^19^ The movement of doctors between centres during training with variation in availability of DP requires additional consideration around the maintenance of skill and confidence (and competence) in reporting GS, an observation relevant not just in the UK.^12^ Furthermore, the FRCPath part 2 examination is on a GS rather than digital format which has the potential to introduce anxiety among those preparing for the examination who may feel ‘out of practice’ with making diagnoses on GS, especially given that many organised courses now use digitised slides, in part driven by the need for remote delivery of teaching during the COVID-19 pandemic.

The results of our survey have been enlightening in that while the trainees overall perceive a large amount of educational benefit in relation to access to DP, particularly during the COVID-19 pandemic, as highlighted by others,^20–22^ there is evidence of variability in the uptake of DP in the diagnostic setting, and a perceived lack of confidence in its routine use.

The reason for the perceived reluctance to fully use DP for diagnostic reporting is not entirely clear from the survey. The respondents showed good awareness of the potential pitfalls in digital diagnosis, in line with those previously reported within the literature,^23^ and generally felt that they had sufficient training to use the digital platform. But levels of confidence in reporting digitally were not uniformly high and only 3 of 10 trainees with diagnostic DP experience were ‘not concerned’ about the accuracy of reporting on DP versus GS. While those using DP diagnostically were aware of the RCPath guidance on validation, a minority had reviewed any of the available validation sets, which is a core component of the governance structures recommended at consultant level during the transition to DP. It could be inferred that the lack of a ‘validation-type’ exercise may have impacted on confidence in digital reporting and a willingness to use the system routinely, confounded by a degree of concern (shown by 4 of 10) about maintaining competence in reporting on GS. Indeed, personal communication with trainees subsequent to the survey indicates enthusiasm for a ‘validation’ process to aide confidence in their diagnostic interpretation on DP, and suggests that the motivation to transition to DP is impacted by wider issues around the need to maintain skills in GS reporting both during training and for future job opportunities at consultant level. It was also highlighted that making the transition to DP during the later stages of training when focus is necessarily on developing confidence in independent reporting is potentially an additional challenge. Consideration of the stage of training at which DP is accessed for diagnostic reporting is therefore also important, although clearly this will be less of an issue when DP is more widely available.

Significantly, the skill to report on GS is required for the FRCPath part 2 examination; however, within our cohort, it appears that transitioning to DP ahead of the examination was not a significant concern as the perceived benefits of rapid access to larger numbers of cases digitally outweighed the reduction in reporting on GS, although there were mixed opinions as to whether having formal teaching and practice examinations during this period in a GS format might be more beneficial. It should be noted for context locally that while DP reporting has been adopted widely within the digitally enabled department, the GS are routinely sent out to the pathologists, and therefore any impact from more limited access to GS for diagnostic cases may not be reflected fully in the current survey.

Maintenance of competence in GS reporting also remains relevant due to the rotational nature of the training programme, given that some centres are currently without access to DP, a situation not unique to our region; and this must be a consideration for training going forward. We would advocate that therefore trainees must be supported to ensure that they feel confident of the return to GS reporting and given the time to make the adjustments.

It was noteworthy that those without current diagnostic DP experience conveyed considerable anxiety about the transition, although it is evident that they have significant DP experience within other contexts such as educational courses. This is important to address, as while positive emphasis on the relevance of *any* DP experience in developing confidence about digital diagnostic reporting may be reassuring, it is noted that 60% (6 of 10) of our cohort accessing DP regarded the clinical standard platform as superior for diagnostic purposes to platforms used purely in the educational setting. This may be a significant consideration if it translates that suboptimal experience with DP within an educational setting negatively impacts confidence in DP.

Going forward, we would advocate a multilayered approach to training within a region with access to diagnostic DP, recognising the overlap with the needs of consultant colleagues but also the specific considerations for training which we have outlined. General education early on in relation to the utility of DP, on practical issues related to integration of DP into the laboratory, and governance-related matters including the validation process, will provide inclusivity across a region with variable access, and can take the form of a group session effectively establishing a ‘community of practice’. Training on technical considerations would be beneficial at the outset to ensure understanding of the functionality of DP and what the limitations may be. Our trainees expressed interest in the ethical and legal considerations in relation to DP, and these needs should be addressed more widely as recent evidence has revealed a general lack of understanding among histopathologists of these aspects.^24^ There should be provision of training on the use of the digital platform, with ongoing support for issues arising. Importantly, we would advocate establishment of a DP validation resource generalised across specialties, enabling trainees to develop confidence in DP reporting. Awareness of existing educational resources developed by early adopters of DP, including those developed by the PathLAKE consortium in the UK (www.pathlake.org), should also be raised. Finally, we recognise that some trainees would benefit from additional support during the transition, and on the basis of the survey results, we have proposed a mentorship scheme whereby trainees with DP experience offer support to others in transition; a scheme that could be rolled out more widely beyond the region as other centres become digitally enabled, and this is to be explored.

## Conclusion

To date, the needs of histopathologists in training as they transition to DP have not been evaluated specifically, and while many of these needs overlap with those of consultant pathologists, there remain specific considerations which are particularly relevant within training regions with variable DP access. We have shown that confidence in reporting on the digital platform is a major factor for consideration, and while there is no formal recommendation for trainees to undergo validation to report digitally, this may be of benefit in aiding successful transition. At this stage where DP is not uniformly available, support is also necessary to maintain skills with GS reporting.

## Data Availability

Data sharing not applicable as no datasets generated and/or analysed for this study.
