# Supplementary material for: Impact of the transition to digital pathology in a clinical setting on histopathologists in training: experiences and perceived challenges within a UK training region
Source: J Clin Pathol. 2022 Jul 29;76(10):712–8. doi: 10.1136/jcp-2022-208416 (PMC10511979; doi:10.1136/jcp-2022-208416)
Supplement: Supplementary data [file jcp-2022-208416supp001.pdf]

Survey of histopathology trainees to assess the impact of the availability of digital pathology for clinical diagnosis within a training region

We are interested in understanding the experiences to date of the histopathology trainees within Thames Valley in relation to the use of digital pathology for diagnostic purposes, and in gauging the opinions of trainees generally on digital pathology and artificial intelligence in our specialty.

We are aware that the experience will be varied across the region and there are questions to explore both the impact of the availability of digital pathology for those who have worked in a centre using digital pathology (Oxford), and for those in centres where digital pathology is not yet available on site. We wish to capture the opinions and experiences across the trainees **in all centres** in region.

The results will be reviewed to identify any areas that could be improved upon, and equally any points of good practice that can be shared more widely. We plan to share the results with you all in due course.

We would therefore be grateful for your time in completing this survey; the survey responses collated will be anonymous.

The results may be used in future peer-reviewed publications and / or presentations.

Thank you in advance for your time.

Questions 1-6: General demographics

Please provide the following details about yourself;

1. Are you a histopathology trainee?

Yes

No (please specify)

2. What is your current level of training?

(please select one of the following options)

ST1

ST2

ST3

ST4

ST5

ST5+

Other (please specify)

3. Which level of the FRCPath examinations are you currently at?

(please select one of the following options)

Pre-FRCPath part 1

Pre-FRCPath part 2 (but post-FRCPath part 1)

Post-FRCPath part 2

Other (please specify)

4. Which of the following best describes your current post?

(please select one of the following options)

Histopathology specialty trainee

Neuropathology specialty trainee

Paediatric pathology specialty trainee

Academic Clinical Fellow (ACF)

Clinical Lecturer / Post-Doc

Non-training post (training level)

Other (please specify)

5. Which of the following best describes your current place of work?

(please select one of the following options)

General hospital

Tertiary/specialist referral centre

Tertiary/specialist referral centre with a research active pathology department

Out of programme currently

Other (please specify)

6. Considering your undergraduate medical education, did you have any experience with digital pathology (virtual microscopy) for histology or histopathology?

(please select all that apply)

No

Yes – images being presented by lecturers on a virtual microscope

Yes – opportunity to use a virtual microscope myself

Yes – other (please specify)

Questions 7-22: General considerations around current level of experience with digital pathology;

7. Have you to date worked in a centre with access to digital pathology for diagnostic reporting?

(please select one of the following options)

No

Yes – I **currently** work in a centre using digital pathology for diagnostic practice (I have < 6 months DP experience)

Yes – I **currently** work in a centre using digital pathology for diagnostic practice (I have > 6 months DP experience)

Yes – **not currently** but I previously worked in a centre using digital pathology for diagnostic practice (I have < 6 months DP experience)

Yes – **not currently** but I previously worked in a centre using digital pathology for diagnostic practice (I have > 6 months DP experience)

8. Which of the following best describes the set-up in your current place of work

(please select one of the following options)

We do not have access to digital pathology

We have a scanner for digitisation of slides but we do not use it at all

We have a scanner for digitisation of slides but it is used for purposes other than diagnostic work (e.g. education, research)

We routinely digitise **some** of our diagnostic slides for digital reporting (but glass slides still routinely sent out to pathologists)

We routinely digitise **all** of our diagnostic slides (histology) for digital reporting (but glass slides still routinely sent out to pathologists)

We routinely digitise our diagnostic slides and do not send out the glass slides routinely from the lab

9. Which of the following activities have you ever used digital pathology for to date?

(please select all that apply)

Diagnostic reporting (any)

For demonstrating cases at an MDT meeting

For my own education (online teaching slides)

For a pathology course (e.g. FRCPath preparation course, ST1 block teaching)

For educating others

For an EQA

For research purposes (excluding clinical trials)

For clinical trial purposes – central pathology review of slides

For clinical trial purposes (FFPE block selection for a trial)

Other (please specify)

None of the above

10. Which of the following best describes your current <u>diagnostic</u> practice in your post?

(please select one of the following options)

I only report using a light microscope with glass slides

I report using a light microscope with glass slides, and occasionally report via digital pathology

I report routinely using both digital pathology and glass slides in roughly equal proportions

I report predominantly using digital pathology

11. If you are <u>not reporting on digital pathology currently</u>, have you had experience of digital pathology reporting for diagnosis previously?

(please select one of the following options, and answer N/A if you are reporting digitally in your current post)

Yes – regular reporting on digital pathology (most cases)

Yes – occasional reporting on digital pathology (some cases)

No

N/A

12. Regarding training to report diagnostic histopathology cases on the digital platform;
(please select all that apply)

I am aware of the RCPath guidance on the validation process for digital pathology

I have undertaken a 'validation process' for diagnostic reporting on the digital platform (**please provide details below in the comments**)

I have reviewed the 'validation' slide sets (some or all) available in my department for the consultants training in digital pathology (or prior department if you have moved job)

I have had training from the vendor (Philips, Leica etc.) on the use of the digital platform

I have attended a training day (of any sort) on digital pathology in clinical practice

I **have not** received any training in diagnostic reporting on a digital platform

Please make any suggestions below as to training that you feel is most useful to aide the transition to digital reporting, including suggestions for the future;

If you have undertaken a 'validation' exercise then please also provide details here.

Questions 13- 21: The following questions are related to your experience of reporting diagnostic cases on the digital platform;

<u>If this does not apply to you as you have not reported any diagnostic cases digitally please answer N/A</u>.

13. Which of the following types of diagnostic cases have you had experience in reporting on digital pathology?

(please select all that apply, and if you have not had experience in reporting diagnostic cases digitally please answer N/A)

Surgical cases (primary reporting)

Surgical referral cases (external referrals)

Educational cases flagged by a consultant / specialty doctor

Educational cases flagged by another trainee

Autopsy pathology

Other (please specify)

N/A

14. If you are reporting on digital pathology, or previously reported digitally, which of the following best describes your use of digital pathology for assessment of diagnostic cases?

(please select one of the following options, and if you have not had experience in reporting cases digitally please answer N/A)

When I report a case digitally I review the case both digitally and on glass slides (all glass slides)

When I report a case digitally I review the case both digitally and on glass slides (selected glass slides)

When I report a case digitally I only review the case digitally and do not review the glass slides (except for problematic areas such as special stains, amyloid)

I only use digital pathology for reviewing diagnostic cases to assess measurements e.g. margins, or for review for assessment of need for levels / immuno / special stains, but otherwise make my diagnoses on glass slides

Other scenario (please specify)

N/A

15. If you have had experience reporting diagnostic cases on a digital platform, how quickly do you feel that it takes to get confident in reporting digitally (in general, as there may still be cases you rather report on glass)?

(please select one of the following options, if you have not you have not had experience in reporting diagnostic cases digitally please answer N/A)

< 1 month

1-3 months

3-6 months

> 6 months

I do not feel confident reporting diagnostically on a digital platform, but feel that I will do in time

I do not feel that I will ever be entirely confident reporting diagnostic cases digitally

N/A

16. Considering your experience in reporting diagnostic cases on digital images rather than glass slides, please answer the following;

(if you have not had experience in reporting diagnostic cases digitally please answer N/A)

Strongly disagree, disagree, neutral, agree, strongly agree, don't know, N/A

I am confident in making diagnoses on digital pathology (generally)

I do not feel that digital pathology for clinical diagnosis is different to diagnosis on glass slides

I find reporting on digital pathology more difficult (generally) than on glass slides

It takes me longer to review diagnostic cases digitally compared with glass (in general)

When reporting a case I usually review the digital slides in preference to the glass slides

I prefer to report digitally than on glass slides

I am concerned about the accuracy of reporting on digital images vs glass slides

I have an awareness of recognised areas of potential pitfall in diagnosis on digital pathology in general

I find that reviewing cases on the diagnostic digital platform (IMS) is superior in terms of ability to make a diagnosis, than reviewing a similar case on a non-diagnostic platform (i.e. for educational use)

17. Considering training and the set-up to report diagnostic cases on digital images vs glass slides, please answer the following;

(if you have not had experience in reporting diagnostic cases digitally please answer N/A)

Strongly disagree, disagree, neutral, agree, strongly agree, don't know, N/A

I feel that I have had sufficient training to report on a digital platform

I **do not** feel that I needed any specific additional training to report diagnostic cases digitally

I have felt supported in the transition to digital pathology

I have an appropriate workstation set-up for digital reporting

I would like to know more about potential challenges / pitfalls in diagnosis on digital pathology

I would like additional training on the use of the digital platform (functionality)

I would like to know more about the integration of digital pathology into the laboratory workflow

I would like to know more about the IT considerations in relation to digital pathology

I would like to know more about the data governance / ethical considerations in relation to digital pathology

I am concerned about maintaining my competence in reporting cases on glass slides

Any comments

18. Can you please provide examples of any aspects of reporting diagnostic cases that are easier in your experience on digital images than on glass slides;

19. Can you please provide examples of any aspects of reporting diagnostic cases that are more challenging in your experience on digital images than on glass slides;

20. Considering your experience in reviewing clinical cases with a consultant histopathologist since the introduction of digital pathology into your workplace, how have these cases been reviewed?

(please select all that apply and if you have not had experience in reporting diagnostic cases digitally please answer N/A)

Reviewing cases on the digital platform **together in real-time** (in person)

Reviewing cases separately on the digital platform **in real-time but remotely** with discussion of the cases via a video-conferencing platform or telephone

Reviewing cases separately but with email communication about the interpretation / diagnosis and any questions

I reviewed the cases on the digital platform but the cases were reviewed together in person on glass

N/A

Other (please specify)

Any comments

21. **Considering your opinion on the educational / training value when reviewing clinical cases with a consultant histopathologist, please answer the following;**

(if you have not had experience in reporting diagnostic cases digitally please answer N/A)

Strongly disagree, disagree, neutral, agree, strongly agree, don't know, N/A

It is educational to review cases on the digital platform **together in real-time** (in person)

It is educational to review cases separately on the digital platform **in real-time but remotely** with discussion of the cases via a video-conferencing platform or telephone

It is educational to review cases separately but with email communication about the interpretation / diagnosis and any questions

It is educational to review cases on the digital platform but then to review the case together in person on glass

I prefer to review the cases with a consultant on the digital platform (in person or remotely)

I prefer to review the cases with a consultant on glass slides (even if I have reviewed them digitally)

I do not think it makes a difference whether the case is reviewed with the consultant on glass or on the digital platform

22. **If you have previously had experience reporting diagnostic cases on the digital platform, but are now working in a centre without access to digital pathology, please answer the following;**
(please select N/A if this does not apply to you)

Strongly disagree, disagree, neutral, agree, strongly agree, don't know, N/A

I have not had any problems with the change to reporting solely on glass slides from digital pathology

I do not feel that the variation in reporting (digital vs glass) is of any real significance

The transition back to reporting on glass slides has been challenging for me

I feel that I lost my confidence on reporting cases on glass slides as a result of previously reporting digitally

Recognition needs to be given to the variability in reporting format (digital vs glass) within a training region

I feel that the variation in reporting across training centres has negatively impacted on my training

Any comments

23. **Please consider the following questions in relation to your training experience since the introduction of digital pathology into your workplace (please try to consider this in the absence of the impact of Covid-19);**

(if you have not been based in a department with access to digital pathology please answer N/A)

Strongly disagree, disagree, neutral, agree, strongly agree, don't know, N/A

The introduction of digital pathology into the department for diagnostic use has been a positive experience

I feel that access to digital pathology in the department has allowed me to see a greater number of cases than I would have expected to in my attachments

I feel that access to digital pathology has facilitated greater case sharing **by the consultants to the trainees**

I feel that access to digital pathology has facilitated greater case sharing **between the trainees**

I feel that access to digital pathology within the department has resulted in less contact for training purposes with consultant histopathologists in general

The introduction of digital pathology into the department for diagnostic use has impacted negatively on my training

Any comments

Questions 24-26 : the following questions are focussed on digital pathology in relation to the FRCPath examinations;

24. **Please consider the following in relation to the FRCPath examinations;**

Strongly disagree, disagree, neutral, agree, strongly agree, don't know, N/A

The availability of digital pathology within our region provides greater opportunity to see clinical cases than I would otherwise expect to have

I feel that I have seen a **greater range** of clinical cases as a result of the availability of digital pathology within region

The utility of digital pathology in a diagnostic setting impacts negatively on preparation to take the FRCPath part 2 examination on glass slides

The utility largely of digitised cases for teaching sessions in region impacts negatively on readiness for the FRCPath examination on glass slides

25. **Have you sat the FRCPath Part 2 examination since commencing digital reporting of diagnostic cases (i.e. since digital pathology has been rolled out within your centre)?**

(please select N/A if this does not apply to you)

Yes

No

N/A

26. <u>If you have sat the FRCPath Part 2</u> (on glass slides), <u>and</u> have been working in a centre with access to diagnostic digital pathology during the preparation period for the exam, please answer the following;
(please select N/A if this does not apply to you)

Strongly disagree, disagree, neutral, agree, strongly agree, don't know, N/A

I **do not** feel that regularly reporting and reviewing cases on a digital platform vs glass slides has any impact on readiness for the FRCPath Part 2 examination

In preparation for the FRCPath Part 2 examination in preference I reported diagnostic cases on glass vs digital

Reviewing my diagnostic cases with a consultant pathologist on glass slides rather than digitally in the period leading up to the FRCPath Part 2 examination would be more helpful

Practice examinations for the FRCPath part 2 are more relevant on glass slides than on a digital platform in the period leading up to the FRCPath part 2 examination

Teaching sessions specifically for preparation for the FRCPath part 2 examination are better on glass slides than on a digital platform

Teaching courses (e.g. for the FRCPath part 2 examination) should utilise glass slides in preference to digital slides

I do not feel that it matters whether educational / teaching cases are on the digital platform or glass slides for the purpose of FRCPath part 2 examination preparation

I feel that access to digital pathology for diagnostic purposes within the centre I was working enabled greater opportunity for me to see cases in preparation for the FRCPath part 2 examination (than I would have had in a department reporting solely on glass slides)

I accessed historic digitised diagnostic cases in my department during the period of preparation for the FRCPath part 2 examination

I accessed historic diagnostic cases **remotely** on the digital platform for preparation for the FRCPath part 2 examination

The ability to access diagnostic cases (for exam preparation) **remotely** on the digital platform has made the exam preparation easier for me

I do not feel that access to digitised slides within my centre has had any impact on my exam preparation

Any other comments

Questions 27-30 : the following questions are related to the impact of the introduction of digital pathology into the region, and for those not currently reporting diagnostic cases digitally, your opinions in relation to digital pathology;

27. **In terms of the impact of access to digital pathology within the region and your training experience, please answer the following;**

Strongly disagree, disagree, neutral, agree, strongly agree, don't know

Until this survey I was not aware that digital pathology was being used within a centre(s) in my training region

I do not feel that having digital pathology installed for diagnostic use within a centre(s) in my training region has any relevance to my training experience

The introduction of digital pathology into the training region has been a positive experience

I feel that access to digital pathology within the region has provided greater teaching / training opportunities across the region

I feel that access to digital pathology has facilitated greater case sharing between the trainees within the region (beyond centre(s) with access to digital pathology)

I feel that the ability to gain experience in region in reporting cases both digitally and on glass will be of benefit to me in the future as a consultant histopathologist

I feel that personal experience with diagnostic digital pathology during training will impact on my future job choices as I am more likely to applying for a consultant post in a centre with access to digital pathology

I would like to see greater use of the digital platform in educational / training events (please provide details below)

Any comments

28. **If you are currently working in a centre <u>without</u> access to digital pathology and <u>have to date not had experience</u> in working in a centre reporting diagnostic cases on a digital platform, please answer the following;**

(please select N/A if this does not apply to you)

Strongly disagree, disagree, neutral, agree, strongly agree, don't know, N/A

I am concerned that the variability in access to digital pathology for diagnostic practice across training centres will impact negatively on my training overall

I do not consider the variability in access to digital pathology across training centres to be an issue in relation to my training experience in histopathology

I feel that access to digital pathology within any centre within the training region has had a positive impact on my training overall

I am apprehensive about the transition to digital pathology in my own practice when this is available to me

I look forward to being able to report diagnostic cases on a digital platform

I do not feel that reporting diagnostic cases on a digital platform will be any different to reporting on glass slides

I feel that digital pathology is over-hyped

29. Please provide up to 3 suggestions as to what you feel will be the most challenging aspects of your own transition from reporting cases on glass slides to reporting digitally;

30. Please provide up to 3 suggestions as to what you feel will be advantages of reporting cases digitally vs on glass slides;

Questions 31-33: finally, the following questions are related to general considerations around the use of digital pathology and artificial intelligence in relation to histopathology.

31. In relation to general considerations around the use of digital pathology, please answer the following;

Strongly disagree, disagree, neutral, agree, strongly agree, don't know

The implementation of digital pathology into diagnostic practice is a positive step for practising pathologists

I think it likely that as a consultant pathologist I will routinely be reporting cases on a digital platform

As a consultant histopathologist I would like to report my cases on a digital platform

I feel that digital pathology is over-hyped and will not last

I have no interest in digital pathology

32. Regarding the potential role for artificial intelligence (AI) in the setting of pathology and your own experience of AI in pathology, please answer the following;

Strongly disagree, disagree, neutral, agree, strongly agree, don't know

I have an awareness of the potential for AI to play a future role in routine diagnostic practice in pathology

I look forward to the potential to use AI in my own practice as a pathologist

I am aware that AI tools already exist that may aide pathologists in **making diagnoses**

I am aware that AI tools already exist that may aide pathologists in **assessing routinely reported prognostic/predictive features in clinical cases**

I believe that AI tools will be able to **derive novel insights** into disease biology and disease prediction/prognosis in the future

I believe that novel features derived by AI will be able to be used in clinical pathways in the future

I am aware that there are already examples of accredited AI tools for use in pathology

I have seen examples of AI tools being used in pathology

I have personal experience of the use of AI in pathology for diagnosis/assessment of histological features

I am interested in the use of AI in pathology

I am worried about the use of AI in pathology

I feel that the role of AI in pathology is over-hyped

I have been involved in research in the development of AI in pathology

I would like to be involved in research in the development of AI in pathology

I am concerned that digital pathology and artificial intelligence will replace pathologists

I have no interest in AI in relation to pathology

33. **If you have seen AI in action in pathology, please can you provide examples (generally what the AI did – do not need specific examples of tools, e.g. AI to count lymphocytes)**

Any further comments
